# Classification and treatment of food impaction

**DOI:** 10.3389/fdmed.2025.1614381

**Published:** 2025-09-12

**Authors:** Wenxi Ma, Jianye Zhou, Bin Ma

**Affiliations:** ^1^ Key Lab Oral Dis Gansu Prov, Key Lab Stomatol, State Ethn Affairs Commiss, Northwest Minzu University, Lanzhou, Gansu, China; ^2^ School of Medicine, Northwest Minzu University, Lanzhou, Gansu, China

**Keywords:** oral orthodontics, food impaction, treatment, prevention, review

## Abstract

Food impaction is a prevalent dental issue where food becomes trapped in interproximal spaces or near restored teeth, leading to discomfort, periodontal disorders, and tooth wear. This review explores the classification and treatment of food impaction, focusing on vertical and horizontal types. Effective management necessitates a thorough examination of the underlying causes to determine the most suitable approach. Vertical impaction is typically addressed through restoring adjacent contacts, adjusting occlusion, reshaping teeth or prostheses, and modifying both adjacent and opposing teeth. Horizontal impaction is managed with periodontal therapy, gingival and papillary reconstruction, addressing dentition deficiencies, and promoting oral hygiene and healthy lifestyle habits. It is crucial to avoid iatrogenic factors as well. Despite advancements in classification and treatment approaches, several limitations persist, including insufficient personalized treatment planning, a lack of longitudinal studies, and inconsistent multidisciplinary collaboration. Future research should prioritize the development of personalized treatment strategies, long-term studies, and the application of innovative technologies to improve treatment outcomes and reduce the incidence of food impaction.

## 1 Introduction

Hirschfeld first introduced the concept of food impaction in 1930, laying the foundation for research in this area. Food impaction occurs when food accumulates in the interdental spaces during mastication, often due to compression and anatomical irregularities. This can lead to periodontal diseases, discomfort, and pain (1). According to the results of the fourth epidemiological survey in China, food impaction is a widespread issue, particularly among individuals with poor oral hygiene and inadequate dental care. Over 70% of elderly individuals aged 55–74 experience conditions such as alveolar bone loss and tooth attachment loss exceeding 4 mm (2). Food impaction is also a common complication following implant fixation, with a notably higher occurrence rate in the posterior dental region compared to individuals with healthy periodontium (3–6). Wong et al. reported that over 40% of patients experienced food impaction after implantation (6), while another study found the incidence rose to 66.9% following single crown implant repairs. Statistics show that food impaction was responsible for 17.5% of unsuccessful fixed denture restorations (7). A longitudinal study found that patients with recurrent food impaction faced a significantly higher risk of developing periodontal disease compared to those without such issues, with an incidence rate of 45% within five years (8).

These findings underscore the importance of addressing food impaction, not only for immediate relief but also for long-term oral health. If embedded food is not promptly treated, it can lead to issues such as bad breath, caries, and soft or hard tissue defects (9), causing significant distress to patients and negatively affecting their quality of life (10). Given the risks associated with food impaction, it is crucial to employ effective classification, diagnostic, and therapeutic approaches. The etiological factors contributing to food impaction include loss of proximal contact (PCL), occlusal discrepancies, malocclusion, positional irregularities, and periodontal recession, among others. Treatment strategies for food impaction vary depending on the underlying cause. Clarifying its classification offers deeper insights into the fundamental mechanisms and influencing factors, which can facilitate the development of more precise treatment protocols.

This review explores various treatment options, including non-surgical interventions, restorative procedures, orthodontic care, and periodontal therapies, with the aim of providing comprehensive and personalized solutions to restore oral health and enhance patients’ quality of life. Additionally, the review seeks to raise awareness of food impaction among dental professionals and patients, emphasizing the importance of oral health and the prevention and management of food impaction-related diseases.

## 2 Classification of food impaction

The classification of food impaction is essential for accurately diagnosing its etiology and implementing targeted treatment strategies. Classifications are typically based on factors such as the underlying cause, the direction of impaction, the extent of the impaction, and the contact relationship between adjacent teeth. However, the complexity of these classification systems has hindered their widespread adoption in the academic field. To address this, some researchers in China have simplified the classification of food impaction into three types—vertical, horizontal, and mixed—based on the direction of the impaction (Table 1). The following sections provide a detailed description of these classifications.

**Table 1:** Different classification of food impaction.

Classification basis	Subtype	Definition/criteria
Etiology-based (11)	1. Loss of proximal contact 2. Occlusal interference 3. Malposition of teeth 4. Morphological abnormality 5. Periodontal breakdown	Categorizes food impaction into five etiological mechanisms with multiple subtypes under each.
Direction-based (12)	1. Vertical 2. Horizontal 3. Mixed	Categorizes food impaction into five etiological mechanisms with multiple subtypes under each.
Extent-based (13)	1. Extensive 2. Partial 3. Limited	Classified by the scope of involved areas. Extensive: ≥ 3 quadrants Partial: 1–2 quadrants Limited: 2–4 teeth
Contact Relationship-based (14)	1. Static 2. Dynamic 3. Mixed	Static: absent or incomplete contact at rest. Dynamic: contact appears normal at rest but separates during mastication. Mixed: combines both.

### 2.1 Classification according to the cause of food impaction

Initially, studies primarily focused on the factors contributing to food impaction. In this context, Hirschfeld classified the mechanisms behind food impaction into five categories based on their etiology, each with its own set of subcategories (15, 16). While the classification system is highly systematic and the subcategories are thorough, it also has some drawbacks. These include its complexity, lack of intuitiveness, and challenges in diagnosis. Clinicians may find it difficult to remember and apply the system, and patients may struggle to comprehend it, which hampers effective communication between doctors and patients and limits its practical use (17).

### 2.2 Classification according to the direction of food impaction

Subsequent studies have provided evidence showing that the direction of food impaction can lead to different clinical outcomes. For example, food that remains trapped in the vertical interproximal spaces for an extended period can exert pressure on the gingival papillae, potentially leading to gingival papillitis. Additionally, the sugars in retained food create an ideal environment for bacterial growth, thereby increasing the risk of proximal caries. As a result, the classification of food impaction based on its direction has gained increasing attention (18).

Food impaction is categorized into three types—vertical, horizontal, and mixed—based on the direction of the food embedding. However, studies by Xu Juan, Fang Bisong, and others have demonstrated that in cases of horizontal food impaction, removing the vertical cause often leads to significant improvement or resolution of the impaction. This supports the hypothesis that primary food impaction occurs in the vertical direction, while horizontal impaction is a secondary phenomenon. Although horizontal food impaction has been reported to account for 9.7% of cases, it is important to note that epidemiological investigation methods are not ideal for classifying food impaction (19). Ultimately, mixed food impaction refers to a combination of both vertical and horizontal impaction.

### 2.3 Classification according to the extent of food impaction

In addition to the previous two methods, Zheng Dize proposed a classification of food impaction based on its extent, dividing it into three categories: extensive, partial, and limited food impaction (20). Extensive food impaction refers to cases where food accumulates across three to four quadrants of the patient's dentition, involving multiple continuous interdental impactions. Limited food impaction, on the other hand, occurs between two to four teeth and is the most commonly encountered type in clinical practice, typically caused by local factors (21). Partial food impaction involves consecutive interdental impactions across one to two quadrants of the dentition.

This classification system is concise, etiologically based, and practical, offering valuable guidance for clinical treatment. However, classifying food impaction solely based on its extent may overlook other important factors and may not adequately address more complex cases. Additionally, this classification is relatively static and does not account for the possibility that food impaction may evolve over time or during treatment. As a result, it provides limited guidance for dynamic assessments of the condition and adjustments to treatment plans. Therefore, further exploration into food impaction classification remains necessary (22).

### 2.4 Classify according to the contact relationship between adjacent surfaces

Food impaction is primarily caused by gaps that develop between the contact areas of adjacent teeth, which can occur either in a static state or during masticatory movements. In this context, the relationship between the adjacent surfaces plays a critical role in the occurrence of food impaction. Based on the characteristics of the contact area gaps at the impaction sites, Fang Bisong et al. classified food impaction into two types: static food impaction and dynamic food impaction (23).

Static food impaction arises when there is a partial or complete absence of proximal contact between adjacent teeth in a non-occlusal state. In contrast, dynamic food impaction is referred to by various names in different studies, such as the proximal close-contact type or the non-anatomical structure disruption type (24). This form of impaction occurs when there is proximal contact between adjacent teeth in a non-occlusal state, but slight separation in the contact area during occlusal movement leads to food entrapment.

There are two primary pathogenesis mechanisms for dynamic food impaction. The first involves a lack of reversible mesial displacement of distal teeth during occlusion, where long-term irreversible mesial displacement of teeth becomes misaligned with adjacent teeth. The second mechanism involves the absence of proper food spillage pathways (25). In clinical practice, mixed food impaction may occur, characterized by both resting space and complex changes during movement. As a result, this classification method may not fully address such situations.

In conclusion, the different classification methods for food impaction each offer specific advantages and limitations (Table 2). Future clinical research should focus on refining these classifications and determining the most effective ways to apply them in practice.

**Table 2:** Advantages and limitations of the classifications of food impaction.

Classification basis	Advantages	Limitations
Etiology-based (26)	Systematic and detailed; covers a wide range of causes.	Complex and less intuitive; difficult for clinicians and patients to grasp.
Direction-based (27)	Simple, intuitive; direction-linked with clinical outcomes (e.g., caries, gingivitis).	Doesn't fully reflect anatomical or etiological complexity.
Extent-based (28)	Clinically practical; useful for treatment prioritization.	Static; ignores dynamic progression and recurrence.
Contact Relationship-based (29)	Addresses both functional and structural problems.	Complex to assess clinically; lacks standardized diagnostic tools.

## 3 Treatment method

It is essential for the dentist to choose the most appropriate treatment strategy for the patient. Traditional treatment options include adjustments, filling procedures, and restorative techniques (30).

Each approach has its own advantages and disadvantages; therefore, managing food impaction requires a thorough evaluation of various factors while selecting an appropriate method tailored to each patient's specific circumstances (31). Employing diverse strategies is crucial to achieving optimal results.

### 3.1 Treatment of vertical food impaction

#### 3.1.1 Resuming contact with neighbors

The management of interproximal contact loss typically involves restoring the optimal interproximal relationship through various restorative methods, such as reconstruction, porcelain addition, or filling adjacent teeth (Table 3). When abnormal interproximal contacts arise due to restoration design flaws (32), manufacturing issues (33), or porcelain chipping (34), the normal contact relationship can often be reinstated by repairing or adding porcelain after removing the faulty restoration.

**Table 3:** Treatment methods for vertical food impaction.

Treatment direction	Subcategory	Specific methods	Indications/notes
Restoring Interproximal Contact (36)	Rebuilding Interproximal Contact New Restoration Resin Filling	Reconstruction, porcelain addition, adjacent tooth filling MDA (Mesiodistal Adjustable) crown Fiber-reinforced chemical-cured resin, flowable resin+ligature	When the original restoration design or porcelain failure causes abnormal interproximal contact Can rebuild contact without replacing the denture Simple, but poor wear resistance and prone to breakage
Adjusting Occlusion (37)	Occlusal Surface Reshaping Occlusal Trajectory Reconstruction Occlusal Force Adjustment	Grinding occlusal surface/ridge edges, reconstructing food escape grooves Deepening/widening grooves, grinding sharp peaks Grinding contact areas, controlling contact area direction	When the contact area is misplaced Increases contact area tightness Allows normal food flow and reduces impaction
Adjusting the Interproximal Zone (38)	Orthodontic Treatment Restoration Adjustment Reducing Contact Area	Adjusting dental position and occlusal relationship Porcelain veneers, full ceramic crowns Grinding contact areas, leaving external gaps	When the contact area is misplaced Increases contact area tightness Allows normal food flow and reduces impaction
Abnormal Tooth/Restoration Shape (39)	Small Defects Large Defects Orthodontic Correction	Resin filling Ceramic restoration Rotation, inclination, impacted teeth	Restores normal tooth shape Good marginal fit and occlusal function Prevents drift of adjacent teeth post-treatment
Adjustment of Adjacent and Opposing Teeth (40)	Adjacent Tooth Mobility Opposing Tooth Interference	Periodontal evaluation, crown restoration, or extraction Periodontal evaluation, crown restoration, or extraction	Requires overall treatment when multiple teeth are loose Relieves opposing tooth pressure and improves occlusion

Recent advancements have introduced the mesiodistal adjustable (MDA) crown, a new solution that allows for the establishment of proper interproximal contact without the need for complete prosthesis replacement. Additionally, interproximal resin fillings offer a viable option for restoring contact when the original restoration remains intact and in good condition (35). For instance, fiber-reinforced chemical curing resins or flowable composite resins, combined with tensioned cerclage wire, have been used effectively to close interproximal spaces, especially in posterior teeth.While these newer resins are easy to manage and cause minimal damage to abutment teeth, they do have certain limitations. Due to their lower filling material content, they are more susceptible to wear and fracture over time, which can compromise their long-term effectiveness.

#### 3.1.2 Adjustment of the occlusal relationship

Adjusting the occlusal relationship is a key approach to eliminating occlusal interference and restoring balance. By reshaping the occlusal surface, marginal ridges, and cusps of the teeth, this process can alleviate food impaction and improve overall function. The adjustment of both the occlusal relationship and the food overflow tract is critical to managing this condition (41). Clinically, two primary techniques are used: regrinding worn pits and grooves and reconstructing the overflow groove to facilitate the flow of food to the buccal and lingual sides. For instance, Ge Jianshui and colleagues conducted jaw adjustment treatments for patients experiencing vertical food impaction with normal posterior tooth alignment. They deepened and widened the proximal middle groove and buccal groove of the distal teeth in patients with maxillary molar impaction. Additionally, they modified the buccal cusp tip and the tongue slope (42). In another study, Xu Juan and others improved food overflow by deepening the developmental groove, which had been penetrated by silicone rubber.

Newell et al. utilized a similar approach by grinding spillways extending to the buccal and lingual sides between the marginal ridge at the impaction site and the adjacent cuspal ridge. They also adjusted the sharpness of the marginal ridge and opposing filling cusps, resulting in symptomatic improvement for 80% of the patients in their study (43). Moreover, adjusting the direction and magnitude of the bite force is essential. When the gap between two teeth exceeds 0.06 mm, the occlusal contact area indicated by the articulator paper becomes excessively large. In such cases, areas showing a deep color should be modified to reduce abnormal occlusal forces (44). Regarding the primary occlusal functional area, occlusal grinding should primarily be focused on the nearest proximal surface to the fovea, with the direction of occlusal force aligned with the long axis of the implant.

#### 3.1.3 Treatment of neighboring contact zones

When food impaction is caused by improper positioning of the contact zone, orthodontic treatment can be employed to adjust the alignment and occlusion of the teeth. Additionally, the contact zone's position and shape can be modified through the use of porcelain teeth or full porcelain crowns (45). In cases where the adjacent teeth are overly tight, grinding adjustments can be made, and the gap-expanding filling method may be applied as part of the treatment.For situations where there is a complete absence of interproximal contact, circular contact bonding along with a movable anti-embedding device made from elastic biomimetic materials can be recommended to address the issue effectively.

When the food impaction arises from a contact area that is too small, restorative options such as resin fillings or porcelain veneers can be used to increase the contact area, improving the tightness between the teeth (46). Conversely, when the contact area is excessively large, it may be necessary to reduce it by adjusting the teeth, thereby restoring the external gap and facilitating food overflow (47). In such cases, it is essential to follow the principle of minimal grinding to avoid unnecessary alterations to the teeth.

#### 3.1.4 Management of tooth or restoration form and position abnormalities

When abnormalities in the shape of teeth or restorations occur, the treatment approach should be tailored to the severity of the defect, aiming to restore the normal shape and prevent further deterioration. For minor defects, composite fillings are an effective treatment option (48). However, for more significant defects, ceramic restorations are preferred due to their superior marginal adaptation and better occlusal function compared to other materials.

For issues related to the positioning of teeth and restorations, such as rotation, inclination, or partial impaction, orthodontic treatment can be used to correct these problems. This typically involves the use of wires and fixed appliances to realign the teeth. Additionally, measures should be taken to prevent adjacent teeth from drifting after extractions (49). If teeth are inclined, orthodontic treatment is necessary to straighten them. In cases of impacted teeth, surgical exposure combined with orthodontic traction techniques can facilitate the eruption of the teeth, reducing the risk of food impaction.

#### 3.1.5 Adjustment of adjacent and opposite teeth

Addressing food impaction requires careful attention to the adjustment of adjacent teeth. Clinical examinations should focus on the morphology of the proximal and occlusal surfaces, as well as the occlusal relationship between adjacent teeth (50). Additionally, if the periodontal health of adjacent teeth is compromised, particularly with excessive loosening, a thorough periodontal assessment and appropriate treatment should be conducted to restore oral health. In cases where a tooth is severely loose and its function cannot be restored, extraction should be considered.

It is common in clinical practice to encounter patients with two or more consecutive teeth that are loosened to varying degrees. For these cases, crowns can be used in conjunction with adjacent teeth, with careful selection of materials and designs to ensure the durability and longevity of the prosthesis. In instances of food impaction caused by opposing filling cusps, modifications to the opposing wedge-shaped cusps and marginal ridges should be made (51). This adjustment not only alleviates pressure on adjacent teeth but also improves the overall occlusal relationship, often preventing further food impaction. However, during the grinding procedure, it is crucial to control the extent of grinding to prevent complications such as tooth sensitivity or damage to the pulp (52).

### 3.2 Treatment of horizontal food impaction

#### 3.2.1 Periodontal treatment

Periodontal treatment plays a crucial role in managing horizontal food impaction. Effective periodontal care typically involves a combination of non-surgical and surgical approaches, aiming to reduce inflammation and restore periodontal health. These treatments not only facilitate tissue healing and enhance therapeutic outcomes by inhibiting bacterial growth but also ensure long-term efficacy, promoting sustained oral health maintenance (57).

When non-surgical methods fail to achieve the desired results, surgical intervention may be necessary. For patients with severe gingival recession and significant periodontal pocket depth, flap surgery can be performed to allow for more effective cleaning and debridement (58). In cases where there is substantial loss of periodontal attachment and bone, gingival graft surgery or periodontal tissue regeneration procedures can be employed to restore the lost tissue, thus reducing the risk of food impaction (Table 4). Furthermore, soft tissue transplantation techniques require careful consideration, as the amount of tissue flap used and the stability of the postoperative flap are critical factors influencing the success of the treatment.

**Table 4:** Treatment methods for horizontal food impaction.

Treatment direction	Subcategory	Specific methods	Indications/notes
Periodontal Treatment (53)	Non-surgical Treatment Surgical Treatment	Scaling, root planing Flap surgery, grafting, regeneration	Early stage of inflammation, safe and long-lasting Significant periodontal pockets and attachment loss
Gingival Papilla Reconstruction (54)	Non-surgical Soft Tissue Grafting Surgical Techniques Personalized Abutment	Temporary abutments, immediate dentures, resin reshaping Autografts, guided tissue regeneration Star-shaped incisions, vertical mattress suturing, etc. CAD/CAM design+full ceramic crowns	Closes “black triangles,” high aesthetics When there is severe papilla loss Preserve/rebuild papilla height during surgery Better marginal adaptability
Tooth Loss Restoration (55)	Dental Implants Fixed Dentures Removable Dentures	Personalized plan, considering bone volume, angle, etc. Bridge+adjacent crowns Removable, suitable for multiple tooth loss	Stable, long-term good outcomes Stable, long-term good outcomes Poor stability, more food impaction
Improvement of Oral Habits (56)	Cleaning Methods Regular Maintenance Smoking Cessation & Diet	Correct brushing, flossing Periodontal check-ups, timely problem handling Stop smoking, supplement vitamins and minerals	Prevents gum recession Improves long-term health maintenance Promotes postoperative healing, reduces complications

#### 3.2.2 Gingival papilla reconstruction

In the reconstruction of the gingival papilla, non-surgical treatments offer a simple, convenient, and minimally invasive solution for restoring soft tissue aesthetics, resulting in minimal patient trauma. For example, temporary abutments and immediate dentures can be used following immediate implantation to facilitate the formation of both anterior teeth and the surrounding gum tissue (59). In cases where a single tooth is missing, modifying the adjacent teeth with veneers or resin shaping can effectively close the “black triangle” and improve the aesthetic appearance around the implant (60).

For patients with significant soft tissue deficiencies, more advanced techniques such as soft tissue grafting and guided tissue regeneration can be employed to restore lost gum tissue, enhancing both the aesthetics and functionality of the gingiva. Preventive measures are crucial for managing gingival papilla insufficiency (61). Throughout the restoration and implantation process, surgeons can employ various techniques such as platform grafting, star incisions, finger splits, middle buccal relaxation incisions, vertical mattress sutures, and oblique mattress sutures (62). These approaches help preserve the gingival papilla and maintain its height. Additionally, CAD/CAM abutments can be used to shape individualized gingival contours, ensuring the papilla's proper form. Research has demonstrated that personalized abutments combined with all-ceramic crown restorations offer superior marginal adaptation and establish a more effective contact relationship (63).

#### 3.2.3 Treatment of missing teeth

Treatment options for missing teeth include dental implants, bridges, and dentures, with the choice depending on the extent of tooth loss and the patient's overall oral health. Dental implants, in particular, have proven to be an effective and reliable solution for filling edentulous spaces (64). Prior to implant placement, a thorough evaluation of the patient's oral health is essential. The planning phase involves careful consideration of factors such as the ideal location, angle, and height for implant placement, as well as the quality and quantity of the alveolar bone and the condition of adjacent periodontal tissue. The distance and height of the peri-implant alveolar crest relative to adjacent teeth are especially critical for achieving aesthetic outcomes, particularly in the anterior maxilla, where the visibility of the gingival margin plays a key role in the overall appearance (65).

When dental implants are not a viable option due to insufficient bone density or other contraindications, alternative treatments like fixed bridges or removable prostheses may be considered. Fixed bridges involve placing crowns on the adjacent teeth to support a pontic (a false tooth) to bridge the gap created by the missing tooth. In contrast, removable dentures are prosthetic devices that can be easily taken in and out of the mouth. They are commonly used when multiple teeth are missing and can be either complete or partial, depending on the extent of tooth loss (66). While dentures offer a functional solution, they may not provide the same stability and comfort as implants or bridges. Patients often report issues with retention, as well as concerns about food impaction beneath the prosthesis, leading to discomfort and complications with oral hygiene.

#### 3.2.4 Develop good oral and lifestyle habits

In addition to surgical and restorative treatments, maintaining excellent oral hygiene is essential for preventing and managing horizontal food impaction. Patients must be educated on proper oral hygiene practices, such as using soft-bristled toothbrushes, dental floss, and avoiding aggressive brushing, which can worsen gum recession (67). Regular periodontal check-ups are also crucial for monitoring gum health and addressing any issues before they escalate.

Lifestyle choices, such as quitting smoking and adopting a healthier diet, play a significant role in supporting oral health. Smoking, in particular, has been linked to delayed healing and an increased risk of complications following dental procedures (68). Patients should be encouraged to follow a balanced diet, rich in vitamins and minerals, to enhance oral health and aid in recovery after any surgical treatment.

### 3.3 Prevention and treatment of implant food implantation

Long-term studies have shown that one of the most common complications following implant treatment is the loss of interproximal contact between the implant and adjacent natural teeth, leading to vertical food impaction. To address this issue, a thorough evaluation of the stress distribution on the remaining natural teeth should be conducted prior to fabricating the implant prosthesis. This assessment aims to prevent the pathological displacement of natural teeth due to interproximal wear during implant use, as well as to avoid the retraction and resorption of both soft and hard tissues surrounding the implant due to excessive bite force (69). Research highlights the importance of maximizing screw retention in the treatment process, as this allows for easier separation of the crown and abutment from the implant. This design facilitates the addition of porcelain between the implant and the natural tooth, making it easier to clean food impaction and effectively address the issue (70). Studies also indicate that recurrence of food impaction tends to occur more quickly following each subsequent repair. Therefore, patients in this category should be scheduled for regular follow-ups every three to six months to ensure careful monitoring and timely intervention.

Treatment of vertical food impaction that preserves interproximal contact after implant placement primarily involves modifying the main occlusal functional area, adjusting the cusp height of opposing teeth, and altering the morphology of both the interproximal and occlusal surfaces of adjacent teeth (71). It also includes adjusting the shape, size, and position of the interproximal contact area of the implant prosthesis. When abnormalities are present in adjacent teeth, orthodontic adjustments may be necessary, and if periodontal conditions are poor, systemic periodontal treatment should be implemented, with extraction of any excessively loose teeth. For the prevention and management of horizontal food impaction following implant-fixed restorations, both patient compliance and iatrogenic factors are critical. Surgeons must ensure every aspect of the implantation process is meticulously controlled. For example, tooth extractions should be performed with minimal disruption to the alveolar bone to preserve the cortical bone (72). Prior to implant placement, a comprehensive evaluation of the patient's periodontal health and the prognosis of the implant restoration should be carried out to ensure the development of a well-considered treatment plan (73).

Recent studies suggest that immediate implantation is beneficial for preserving interdental papillae. Prolonged tooth loss can lead to resorption of the surrounding alveolar bone and gingival recession, which complicates the reconstruction of interdental papillae and increases the likelihood of food impaction. Additionally, the implant superstructure abutment can be designed with a broad, flat contact shape, positioning the contact point near the gingiva. This helps maintain the fullness of the interdental papillae and prevent horizontal food impaction (74). The treatment for horizontal food impaction is similar to that for vertical food impaction, incorporating periodontal therapy, soft tissue grafting, guided periodontal tissue regeneration, the use of artificial gingiva, and removable anti-impaction devices.

### 3.4 Prevention and treatment of iatrogenic factors

A key contributor to food impaction is the medical aspect of dental care. To effectively manage this issue, several steps should be taken. First, improperly executed dental restorations must be promptly corrected or rebuilt to ensure accurate and proper contact points between adjacent teeth. These contact areas should not be excessively tight, as this can harm the gums, nor should they be too loose, as this creates spaces where food can easily become trapped. Effective prevention relies on careful planning and modification of these contact zones (75). When issues arise with fillings, it is crucial to promptly refill or adjust their alignment with neighboring teeth to ensure smooth edges and appropriate height. Smooth edges minimize gum irritation and prevent food particles from adhering, while the correct height supports proper occlusion and reduces the risk of impaction (76).

During orthodontic treatment, plans should be tailored to each patient's specific needs, with the use of gap retainers to maintain proper spacing between teeth, thereby reducing the likelihood of food impaction. In addition, reinforcing oral hygiene practices is essential in reducing the risks associated with food retention (77). To stabilize teeth and improve the health of periodontal tissues, comprehensive periodontal treatments should be employed. These treatments focus on restoring both the structure and function of the teeth, modifying the relationships between neighboring teeth, and maintaining spacing with the help of gap retainers, all of which help prevent food impaction and tooth movement (78). In conclusion, addressing food impaction related to medical conditions requires personalized treatment plans tailored to the underlying causes. Successful intervention can significantly reduce food retention, restore the relationships between adjacent teeth, and ultimately improve oral health and patient comfort.

## 4 Conclusion

The classification of food impaction has evolved over time, with various types displaying distinct etiological factors and clinical manifestations. This progression has laid the groundwork for more targeted treatment strategies. The etiology of food impaction is multifactorial and remains incompletely understood. It involves a range of potential contributing factors, including dental anatomy, occlusal relationships, gingival conditions, and chewing habits, making diagnosis and treatment particularly challenging (79). While periodontal disease and dental caries are recognized as major contributors, food impaction can lead to a cascade of complications such as periodontal lesions, gingival recession, and further caries. These conditions often interact, exacerbating one another.

One significant barrier to effective treatment is the failure to consider individual anatomical and physiological variations that may influence food impaction. This oversight often leads to the adoption of a universal treatment approach, which may not be suitable for all patients. Additionally, the efficacy and long-term durability of existing treatments require further enhancement. Despite a variety of treatment options, some patients continue to experience recurrent food impaction, especially in more persistent cases, where current treatment standards and predictive assessment methods remain unclear (80). Moreover, most research tends to focus on short-term outcomes, which may not provide an accurate picture of treatment efficacy over time.

Beyond the physical discomfort, food impaction can have significant psychological and social effects on patients, such as anxiety related to eating and social interactions. These aspects are often neglected during clinical assessments. Identifying and addressing potential contributing factors during treatment monitoring is critical for preventing and alleviating patient distress. Once the underlying etiology is established, an individualized treatment plan should be formulated, tailored to the patient's specific circumstances and the complexity of their food impaction. Early intervention is crucial to avoid more serious complications (81). In cases where the condition is severe and the treatment is complex, a multidisciplinary approach involving orthodontics, prosthodontics, and surgery is necessary, and consultations with specialists from each field should be sought (82).

With continuous advancements in dental technology and emerging research, many scholars are now utilizing computer-aided systems to study the pathogenesis and treatment of food impaction (83). For example, Zhang Li et al. demonstrated that using the 3Shape intraoral scanner to obtain anatomical and occlusal data can generate digital models for analyzing occlusal contact distribution and intensity (84). This method is simple and intuitive, offering valuable guidance for clinical adjustments and reconstructions. Mustafa assessed the accuracy and realism of the CHA-SB system, which uses a healing abutment scanning body to capture data from various areas of the maxilla (85). Meanwhile, Attar introduced personalized restoration plans via a computer-coded healing abutment system, designing gold-plated titanium abutments that align with the patient's anatomical contours, thereby reducing the risk of food impaction. Additionally, Sui Huaxin et al. developed a mandibular motion simulation system, and Solaberrieta et al. created a digital facebow to simulate bite dynamics, enhancing precision in arch wire adjustments (86).

With the rise of digital dentistry, technologies like CAD, intraoral scanning, and simulation systems are playing an increasingly important role in the diagnosis and management of food impaction (87). These tools improve the accuracy of occlusal assessments and help create personalized treatment plans by allowing better visualization of anatomy and jaw movements. This reduces the risk of complications after treatment. Patient-specific digital abutments and dynamic occlusion analysis have further improved the precision of prosthetic and restorative procedures, leading to better long-term outcomes (88). However, most current studies are limited to small case reports or lab-based research, lacking broad clinical validation. To bring these innovations into routine practice, future research should take a more comprehensive approach (89). This includes studying the causes of food impaction, refining classification systems, and creating standardized treatment guidelines based on long-term clinical data. It is also important to assess patient satisfaction, treatment costs, and the impact on quality of life.

Future research must delve deeper into the classification, underlying mechanisms, and diverse treatment strategies for food impaction. This comprehensive approach should focus on the development of personalized treatment plans, the conduct of longitudinal studies, and fostering multidisciplinary collaboration, all while exploring innovative technological advancements (90). Given the complexity of food impaction, clinicians must maintain a thorough understanding of the factors influencing its occurrence, both before and during treatment. Such awareness will help mitigate risks and optimize therapeutic outcomes throughout the treatment process. Lastly, maintaining optimal oral hygiene and health is essential in preventing and managing food impaction (91). Healthcare providers should educate patients on the importance of interdental cleanliness. By enhancing patients' understanding of food impaction and its consequences, this educational intervention can significantly improve both their prognosis and overall oral health outcomes (92).
